# Exacerbations in Chronic Obstructive Pulmonary Disease: Identification and Prediction Using a Digital Health System

**DOI:** 10.2196/jmir.7207

**Published:** 2017-03-07

**Authors:** Syed Ahmar Shah, Carmelo Velardo, Andrew Farmer, Lionel Tarassenko

**Affiliations:** ^1^ Nuffield Department of Clinical Neurosciences University of Oxford Oxford United Kingdom; ^2^ Institute of Biomedical Engineering, Department of Engineering Science University of Oxford Oxford United Kingdom; ^3^ Nuffield Department of Primary Care Health Sciences University of Oxford Oxford United Kingdom

**Keywords:** COPD, disease exacerbation, mobile health, self-management, pulse oximetry, respiratory rate, clinical prediction rule, algorithms

## Abstract

**Background:**

Chronic obstructive pulmonary disease (COPD) is a progressive, chronic respiratory disease with a significant socioeconomic burden. Exacerbations, the sudden and sustained worsening of symptoms, can lead to hospitalization and reduce quality of life. Major limitations of previous telemonitoring interventions for COPD include low compliance, lack of consensus on what constitutes an exacerbation, limited numbers of patients, and short monitoring periods. We developed a telemonitoring system based on a digital health platform that was used to collect data from the 1-year EDGE (Self Management and Support Programme) COPD clinical trial aiming at daily monitoring in a heterogeneous group of patients with moderate to severe COPD.

**Objective:**

The objectives of the study were as follows: first, to develop a systematic and reproducible approach to exacerbation identification and to track the progression of patient condition during remote monitoring; and second, to develop a robust algorithm able to predict COPD exacerbation, based on vital signs acquired from a pulse oximeter.

**Methods:**

We used data from 110 patients, with a combined monitoring period of more than 35,000 days. We propose a finite-state machine–based approach for modeling COPD exacerbation to gain a deeper insight into COPD patient condition during home monitoring to take account of the time course of symptoms. A robust algorithm based on short-period trend analysis and logistic regression using vital signs derived from a pulse oximeter is also developed to predict exacerbations.

**Results:**

On the basis of 27,260 sessions recorded during the clinical trial (average usage of 5.3 times per week for 12 months), there were 361 exacerbation events. There was considerable variation in the length of exacerbation events, with a mean length of 8.8 days. The mean value of oxygen saturation was lower, and both the pulse rate and respiratory rate were higher before an impending exacerbation episode, compared with stable periods. On the basis of the classifier developed in this work, prediction of COPD exacerbation episodes with 60%-80% sensitivity will result in 68%-36% specificity.

**Conclusions:**

All 3 vital signs acquired from a pulse oximeter (pulse rate, oxygen saturation, and respiratory rate) are predictive of COPD exacerbation events, with oxygen saturation being the most predictive, followed by respiratory rate and pulse rate. Combination of these vital signs with a robust algorithm based on machine learning leads to further improvement in positive predictive accuracy.

**Trial Registration:**

International Standard Randomized Controlled Trial Number (ISRCTN): 40367841; http://www.isrctn.com/ISRCTN40367841 (Archived by WebCite at http://www.webcitation.org/6olpMWNpc)

## Introduction

Chronic obstructive pulmonary disease (COPD) is expected to become the fourth leading cause of death by 2030 [1], imposing a high socioeconomic burden worldwide [2]. It is a chronic, progressive condition caused by airway infection due to smoking or long-term exposure to pollutants (eg, dust, fumes, poor air quality) irritating lungs. Common symptoms of COPD include coughing, chest tightness, breathlessness, fatigue, dizziness, and wheezing. Patients with COPD suffer from “exacerbations,” a sustained worsening of symptoms often leading to hospitalization. In the United Kingdom, COPD exacerbations are now the leading cause of hospitalization, accounting for 15.9% of all hospital admissions [3]. There is growing evidence to suggest that increased frequency of exacerbation episodes leads to faster decline in lung function, reduced quality of life, greater numbers of hospital admissions, and increased health care cost [4]. Exacerbation is thus widely used as a major outcome in clinical studies for patients with COPD, with increasing efforts to try and reduce them. These efforts are hampered by the wide variation in the definitions of exacerbation used in clinical studies, with some based on symptoms (eg, increased breathlessness, sputum volume/color), some on events (eg, requiring hospitalization, taking antibiotics, and/or steroids), and some using a combination of both symptoms and events [4]. Symptoms are typically reported by the patients themselves using a paper-based diary and hence their recording depends on a subjective assessment. Poor adherence to protocol and data validity issues in clinical studies are often cited as major disadvantages of this approach [4]. Event-based definitions, on the other hand, are based on assessment by health care professionals (HCPs) after contact with them has been made by the patient. The disadvantage here is the low level of reporting to HCPs, with as many as 50% of exacerbations being unreported [5]. Until recently, exacerbations could only be identified from patient interviews or from analysis of records of treatment in health care databases or of symptom diary cards filled manually by patients [6].

Telemonitoring allows patients to record their symptoms in real time. Symptom data can then be stored and transmitted, with instant access possible for HCPs. As a result, there have been a number of attempts to use telemonitoring solutions to help manage patients with COPD through early detection of deterioration [7]. However, low compliance, lack of consensus on what constitutes an exacerbation, the small number of patients in most studies, and the relatively short periods of time during which monitoring takes place (less than 6 months) are limitations of COPD telemonitoring interventions [7]. In order to address these challenges, we developed the EDGE (Self Management and Support Programme) digital health system, a telemonitoring platform first evaluated during a cohort study [8] and subsequently used for 12 months, in a randomized controlled trial (RCT), by 110 patients in the intervention group [9]. The EDGE digital system allows patients to report their symptoms directly on an Internet-enabled tablet computer with a user-friendly interface, thereby eliminating most data validity issues. In the RCT, patients were expected to manage their use of relievers, antibiotics, and steroids and record it as part of the symptom diary on the tablet computer. Medication usage data linked to the symptom data were thus available, and so events could be defined based on medication data without the requirement of contact with an HCP. The system was intuitive and easy to use leading to high patient compliance and the collection of large amounts of COPD patient data [9].

Exacerbations are characterized by a number of different scenarios: for example, a patient’s medication changes but the symptoms do not change; a patient's symptoms change, but the intake of medication does not change; or the patient’s symptoms get worse without the patient taking any medication and there is no change for a few days before the patient eventually takes his or her medication. A systematic approach to address such a complex problem is to model it using a finite-state machine (FSM). Assuming that exacerbations can be correctly identified using such an approach, the next step is to investigate if a robust algorithm can be developed to predict exacerbation episodes. This is especially challenging because the protocol for the RCT allowed patients to self-monitor and record their symptom and medication data at a time of their choosing. There were periods, for some patients, during which they self-monitored less frequently and other periods with more frequent data but for different times of the day. Any predictive algorithm must be robust to such irregular sampling and take the variable timing of data recording into account.

In previous work, we developed an algorithm using the EDGE cohort study data, with a limited definition of exacerbation—based on self-reported medication usage, self-reported symptoms, and pulse oximetry data [10,11]. We did not differentiate between correlation and prediction, as the entire period, both before and during an exacerbation, was considered. Ideally, any predictive algorithm developed should identify periods likely to be associated with an impending exacerbation and differentiate them from periods where a patient’s condition is stable (including periods of sustained high medication usage).

## Methods

### Overview

This section first describes the EDGE digital health system used to collect data from the 110 patients in the intervention group during the RCT. Next, the FSM model for identifying exacerbation episodes from symptom and medication usage data is introduced. This is followed by a description of the procedure for extracting “stable” and “prodromal” periods to provide the training data for an exacerbation prediction algorithm relying on robust features. Finally, the classification and validation methodologies adopted are presented.

### Self Management and Support Programme System

The EDGE system is a digital health system with an Internet-enabled tablet computer at its core to support self-monitoring and self-management. The main components include a customized application developed for patients with COPD in collaboration with clinicians and patients, a Bluetooth-enabled pulse oximeter, and a secure back-end server. The application includes several self-monitoring modules, including an interactive symptom diary with a series of questions related to COPD symptoms and to medication usage. A “session” consists of completing a symptom diary questionnaire and a brief recording with a pulse oximeter using a finger probe and typically lasts about 100 seconds [8]. The interactive diary questionnaire allows patients to self-report whether their symptoms have improved, become worse, or not changed compared with their understanding of what is “usual” for them. Chest tightness, breathlessness, sputum volume, and purulence are treated as “major” symptoms, while having a cold or sore throat, and an assessment of feeling generally run down, are treated as “minor” symptoms. Patients also report whether they are taking relievers, antibiotics, or steroids or any combination of the 3.

### Finite-State Machine Model for COPD Exacerbation

According to the World Health Organization (WHO) and US National Heart, Lung, and Blood Institute Global Initiative for Chronic Obstructive Lung Disease (GOLD), an exacerbation is defined as “an event in the natural course of the disease characterized by a change in the patient’s baseline dyspnea, cough, or sputum that is beyond normal day-to-day variations, is acute in onset, and may warrant a change in regular medication in a patient with underlying COPD.” Although the definition is comprehensive, it raises a number of questions (eg, what are “normal day-to-day variations”?), thereby limiting its adoption as a practical definition in the context of clinical studies.

An FSM is a mathematical abstraction extensively used in the domain of logic design [12]. In FSM models, there are a finite number of “states” and a machine can transition from one state to another depending on “inputs.” In this context, the “machine” will model a patient, and “inputs” are changes in symptoms and/or medication uptake. A patient will be in a particular “state” at any given time. Depending on changes in symptoms or medication usage, the patient’s state can transition to another state. In addition to a “normal” state and an “exacerbation” state, we define a “transitional” state to account for those situations in which the patients are not in their normal state but their condition is not poor enough for them to be considered as having an exacerbation.

As already mentioned, there are a large number of exacerbation definitions. The following definition of exacerbation, based on clinical experience and a comprehensive review of the literature, was adopted by the clinical experts running the EDGE RCT: “An exacerbation is defined as a change in medication or an HCP contact (hospital admission or any documented contact, face-to-face contact or telephone calls) in the presence of a significant increase in symptoms.”

“Change in medication” or “medication event” is defined as (1) increased use of reliever inhaler for at least 48 hours and (2) starting oral steroids and/or antibiotics. “Significant increase in symptoms” is defined as the presence of at least two symptoms, one of which should be a major symptom.

**Figure 1:**
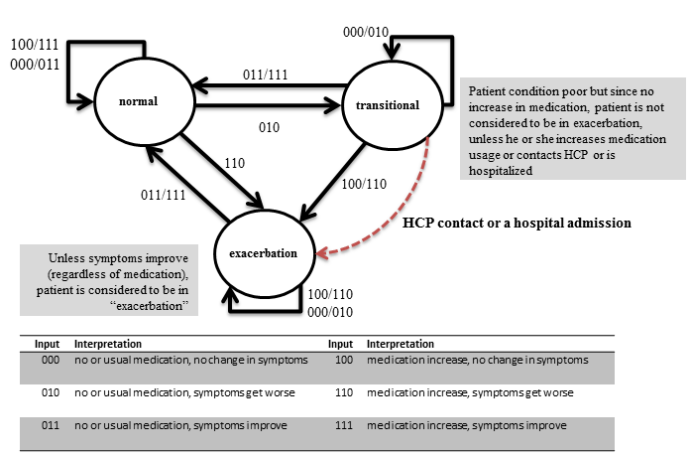
A finite-state machine to model chronic obstructive pulmonary disease patient condition and its changes depending on symptoms and medication usage. HCP: health care professional.

The 3 states in the FSM-based model are “normal” (patient is completely normal), “transitional” (patient’s symptoms have become worse but no medication event or hospital admission or contact with HCP), and “exacerbation.” Completion of a new symptom diary questionnaire generates a new set of inputs. Figure 1 shows the FSM model with its 3 states and the transitions between them depending on the 3-bit “inputs” encoding different combinations of symptoms and medication usage. The first bit encodes medication usage (0 = no medication or usual medication; 1 = medication event, as defined above). The second and third bits encode the status of the self-reported symptoms (00 = no change in symptoms; 10 = symptoms get worse; 11 = symptoms improve). It can be seen from Figure 1 that patients stay in the normal state regardless of whether they take medication or not, unless their symptoms get worse. Once a patient’s symptoms get worse, he or she either transitions to a transitional state or into the exacerbation state, depending on whether there is a change in medication or medication event, as defined above. In the transitional state, a patient returns to the normal state if his or her symptoms improve (regardless of whether a medication event occurs or does not occur). Whenever the symptoms have not improved, a transition to the exacerbation state occurs instead if there is a medication event, or if the patient is hospitalized or makes contact with an HCP (regarding his or her COPD symptoms). In the exacerbation state, a patient only transitions back to the normal state if his or her symptoms improve. The definition of symptom improvement is a mirror image of the definition of “symptoms getting worse” given above: at least two symptoms, one of which must be a major symptom, must get better in comparison with the data recorded in the previous diary session.

### Selection of Independent Periods

In order to develop algorithms for prediction of exacerbation episodes, it is important first to identify the periods of time (in days) during which a patient’s condition is stable, as well as periods of time when a patient’s condition can be considered to be deteriorating before an exacerbation event. A previous study [13] has suggested that symptoms tend to worsen during the 7 days immediately before an exacerbation episode. We therefore define a time window of 7 days before an exacerbation event and label all such instances in our dataset as “prodromal” periods. Similarly, “stable” periods are defined as 7-day periods during which a patient must be in the normal state throughout. These periods have a 7-day “guard band” at the start and the end of the period. This is to ensure that, for every stable period, there are at least 7 days at the start of the stable period since a transitional or exacerbation state ended and at least 7 days at the end of the stable period before the next transitional or exacerbation state occurs.

**Figure 2:**
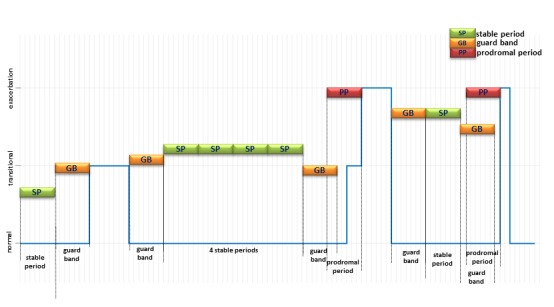
Identification of “stable periods” and “prodromal periods” over the course of monitoring of a single patient. Notice that there are only 4 stable periods identified between the time when the patient returns to normal from the transitional state and the time when the patient goes from normal to the transitional state. There is only 1 stable period after the patient returns to normal after the first exacerbation and before the second exacerbation occurs. In this illustration, each vertical line represents a single day, and “stable,” “prodromal,” and “guard bands” are all 7 days long.

Figure 2 illustrates the identification of “stable” and “prodromal” periods for a single subject who was in different states over the course of the trial.

### Vital Signs From Pulse Oximetry

Commercial pulse oximeters typically provide only oxygen saturation (SpO_2_) and pulse rate measures. However, it is also possible to extract respiratory rate using the waveform—called photoplethysmogram (PPG)—recorded by a pulse oximeter with an appropriate algorithm. Briefly, respiratory rate modulates both the amplitude (primarily due to the mechanical effect of breathing) and frequency (due to respiratory sinus arrhythmia) of the PPG [14]. In the analysis presented in this paper, we focus on extracting respiratory rate from the amplitude modulation of the PPG because the phenomenon of respiratory sinus arrhythmia is reduced both with age [15] and pharmacological interventions [16]. The method used for estimating respiratory rate from the PPG waveform was based on computing the median frequency spectrum from a number of autoregressive models of the 30-second waveform segments recorded during the self-monitoring with the finger probe pulse oximeter. This novel signal processing approach was developed by us to estimate respiratory rate from PPG data in a different clinical setting [17]. Estimation of the respiratory rate from the amplitude modulation of the PPG requires a preprocessing stage in which an appropriate low-pass filter attenuates as much high-frequency content (corresponding to the cardiac frequency) as possible while preserving the low-frequency content (corresponding to the respiratory frequency). Because the work described in [17] was based on a population of pediatric patients with different values of respiratory and heart rates, the preprocessing stage was modified to take into account the lower values of respiratory and heart rates in the adult population. The cutoff frequencies of the transition band of the low-pass filter were varied according to the heart rate of the patient in a given session (ie, from 0.5 × heart rate to 1.2 × heart rate). Consequently, for each recording session from a participant in the RCT, 3 vital signs (heart rate, SpO_2_, and respiratory rate) were obtained from the 30-second pulse oximetry data.

### Feature Extraction

The labeling shown in Figure 2 is used to create a database of vital sign data from 7-day stable periods and 7-day prodromal periods (ahead of the exacerbation events). This database will enable us to construct a 2-class classifier, with the vital sign data as its inputs, to assign the data to either the stable or prodromal class. Classification of a 7-day period as prodromal is of course equivalent to exacerbation prediction.

**Figure 3:**
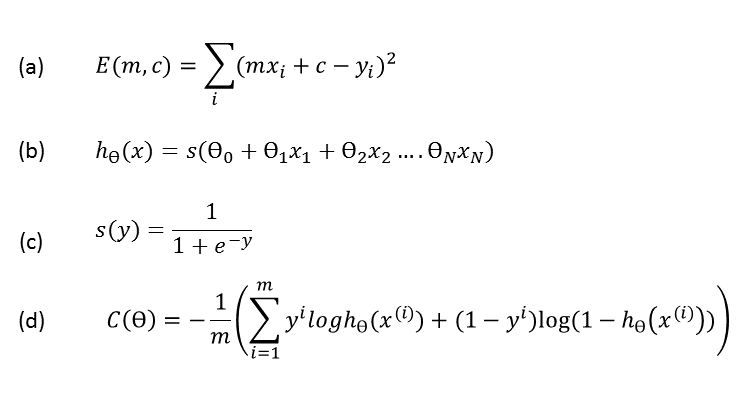
(a) Least Squares Fitting to determine the gradient and y-intercept in each 7-day period(b) Hypothesis function (c) Sigmoid function(d) Cost function for logistic regression.

It is important to capture the time course of each vital sign during these 7-day periods. A number of traditional algorithms developed for time series analysis cannot be directly applied because of the irregular number of recordings over a given period, both within and across subjects. Rather than applying approximations, for example, grouping multiple recording sessions within a day together (a rare occurrence), or imputing missing data (a much more common phenomenon), we fit a straight line, using a least squares criterion, to each set of vital sign data in each period. This corresponds to finding the minimum of *E* (*m,c*) defined in equation (a) in Figure 3, where *m* is the gradient, *x*_i_ is the time at which the *i*^th^ session was recorded in a given 7-day period, *y*_i_ is the corresponding value of the vital sign, and *c* is the y-intercept value. For each vital sign in each 7-day period, the minimization of equation (a) will provide the gradient, *m*, whose magnitude will reflect whether the value of the vital sign is increasing, decreasing, or not changing, and an intercept value that is dependent on both the mean value and the gradient.

**Figure 4:**
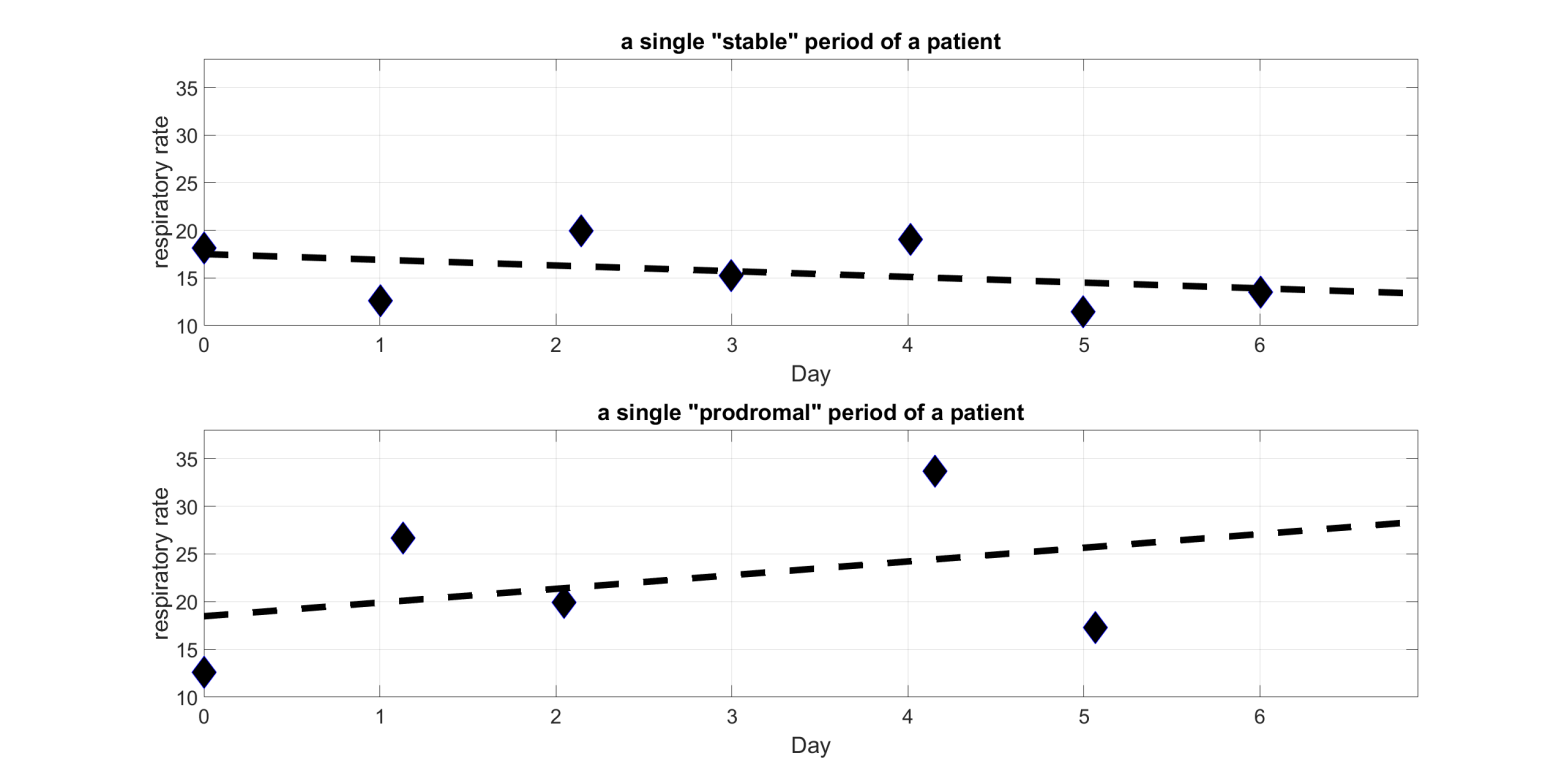
Illustration of straight line fitting during a “stable” period (gradient: −0.60, y-intercept: 17.5) and a “prodromal” period (gradient: 1.43, y-intercept: 18.47).

Each 7-day period is normalized in time with respect to the first self-monitoring session in that period (the first session in each 7-day period is taken to be at time 0). Figure 4 illustrates this for 2 cases, a “stable” period and a “prodromal” period. In this particular illustration, it can be seen that the patient in the stable period had a higher mean respiratory rate, which was gradually decreasing during the 7 days. The patient in the prodromal period has a lower mean respiratory rate but there is a clear increase over the 7 days. With this simple linear fit method, we capture the trend of each vital sign over the 7 days. Periods with irregular self-monitoring times or missing data can still be analyzed.

### Classification and Validation

A logistic classifier was applied to the vital sign feature vectors derived from both the stable and prodromal periods in order to investigate the discriminatory power of these features for classification (and hence exacerbation prediction). Equation (b) in Figure 3 gives the hypothesis function used in logistic regression where *x*_n_ and θ_n_ are the *n*^th^ feature and parameter, respectively, and *s* (*y*) is a sigmoid function (equation (c) in Figure 3). The output of the sigmoid function is bounded between 0 and 1 and can thus be interpreted as a probability or, in this case, the likelihood of a patient’s condition deteriorating and leading to exacerbation (the prodromal period). The mean and gradient values (*x*_n_) of each of the 3 vital signs are the inputs to the logistic classifier.

The cost function given by equation (d) in Figure 3 outputs a large value when the predicted value *h*_θ_(*x*), is very different from the true class value, *y*, and a very small output when the predicted value is close to the true class value. An additional benefit of *C* (θ) as a cost function is that it is convex and thus a global minimum will always exist, which can easily be determined with a gradient descent algorithm.

Correct classification of a prodromal period was deemed to be a true positive and correct classification of a stable period was deemed to be a true negative. Incorrect classifications of prodromal periods and stable periods were taken to be false negatives and false positives, respectively. To test the generalization capability of the classification algorithm, we used 10-fold cross-validation. The data from both classes were randomly divided into 10 folds, with 9 folds used for training and 1 fold for testing. This was repeated 10 times, each time using a different fold for testing. This allowed us to obtain classification results on the whole dataset. Because the partitioning of data into 10 folds is random, the process of applying 10-fold cross-validation was repeated 1000 times, thereby allowing us to find the possible range of classifier performance. For performance evaluation, the receiver operating characteristic (ROC) curve was determined for each iteration and the mean ROC curve along with the 95% confidence interval was then computed. We then estimated the area under the curve (AUC) to compare the different classifiers.

## Results

### Dataset

Table 1 provides summary statistics on the number of days, sessions, and compliance of the 100 patients in the RCT intervention group with near-complete symptom diary and pulse oximetry data. On average, a patient self-monitored for 354 days, with a minimum usage of 5 days every week throughout this period.

**Table 1:** Overall number of days, sessions, and compliance of 100 patients in the EDGE (Self Management and Support Programme) study with both symptom diary and pulse oximetry data.

Metric	Across all patients	Per patient	
		Mean (SD)	Range
Number of days in study	35,439	354 (31)	194-403
Number of sessions	27,260	273 (68)	61-373
Number of days system used	27,074	271 (67)	60-373
Mean compliance (days/week)		5.3 (1.2)	1.8-6.9

### Identification of Exacerbations

**Figure 5:**
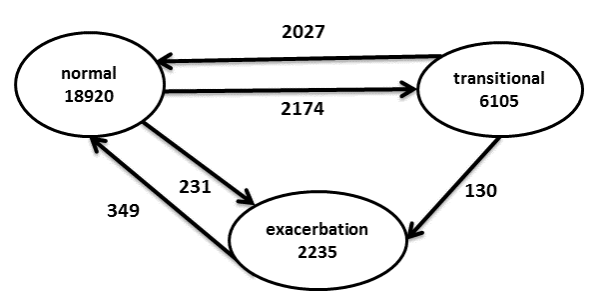
Summary of state transitions illustrating the total number of sessions in each state (inside the circles) and the number of instances of each transition between states across all patients (numbers next to the arrow between states) in the randomized controlled trial.

There were a total of 361 exacerbation events after applying the proposed FSM model to the 27260 sessions from 100 patients. Figure 5 summarizes the total number of sessions in each state (given inside the circles), as well as the number of times that a patient transitioned from one state to the other (given next to the corresponding arrow). As expected, patients were mostly in the normal state (18,920/27,260, 69.41% of sessions completed) and were in the transitional state for 22.40% (6105/27,260) of the sessions. In 2174 instances, patients in the normal state entered the transitional state as a result of their symptoms deteriorating but without increasing their medication. In 93.24% of these cases (2027/2174), their symptoms subsequently improved and they returned to the normal state. For 97.08% of the 2027 cases (1968/2027), this happened without any medication increase; in only 2.91% of the cases (59/2027) did patients increase their medication before returning to the normal state. In the cases for which patients did not increase their medication while returning to normal state, the mean and median number of days in the transitional state were 3.2 days and 1.8 days. In the cases for which patients increased their medication while returning to normal state, the mean and median number of days in the transitional state were 4.2 days and 3.0 days.

Only 2235 sessions (8.20% of total sessions, 2235/27,260) were completed while the patient was in the exacerbation state. In 64.0% of cases (231/361), a patient’s state transitioned directly from the normal to the exacerbation state, corresponding to patients increasing their medication as soon as their self-reported symptoms got worse.

All patients were assumed to be in the normal state at the start of the RCT, but only 71 patients ended up being in the normal state at the end of the study, with the remaining 29 patients ending in either the transitional state or the exacerbation state. Thus, there were 29 more transitions out of the normal state (2405) than into the normal state (2376) and also 17 and 12 extra transitions into the transitional and exacerbation states, respectively.

### Distribution of Exacerbations

**Figure 6:**
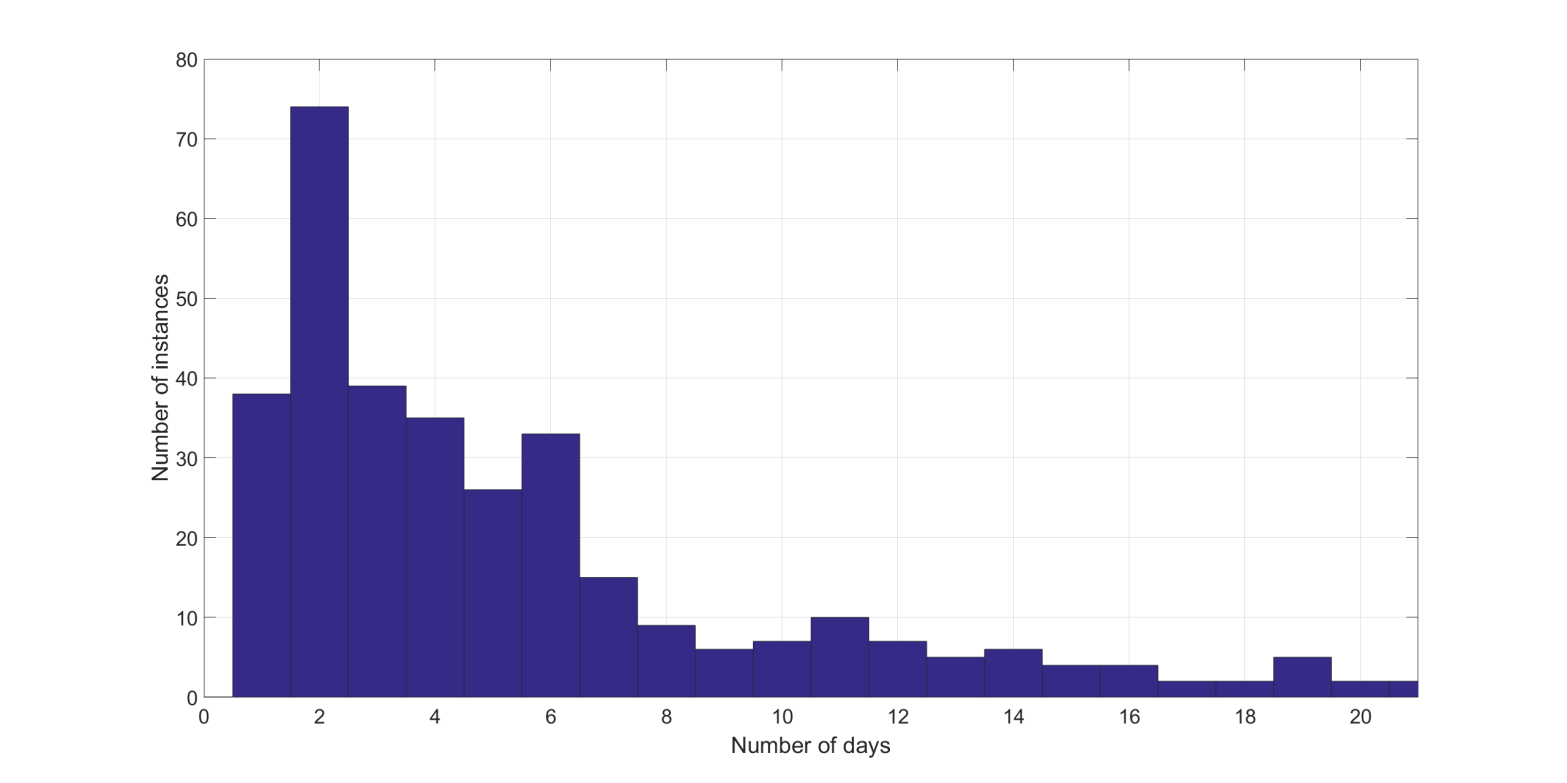
Distribution of exacerbation length (only those with episodes lasting 21 days or less are shown).

Table 2 and Figure 6 illustrate the distribution of length of exacerbation episodes. It can be seen that most of the exacerbation episodes lasted for up to 7 days (260/361, 72%). However, there was considerable variation in the length of an exacerbation episode across the population, with a mean length of 8.8 days and a median length of 4 days. On the basis of the total number of monitoring days, patients were in the exacerbation state for about 9% of the time (3180/35,439 monitoring days).

**Table 2:** Length of exacerbation events across all patients (91.7% of exacerbation episodes, 331/361, were within 21 days).

Metric	Number
Total number of events	361
Event length 1-7 days, n (%)	260 (72.0)
Event length 8-14 days, n (%)	50 (13.9)
Event length 15-21 days, n (%)	21 (5.8)
Mean length of exacerbation, days	8.8
Median length (interquartile range) of exacerbation, days	4 (2-9)

### Characteristics of Vital Signs During Stable and Prodromal Periods

Table 3 summarizes the total number of periods and the corresponding number of sessions, as well as summary statistics for the various features extracted for each of these periods, both stable and prodromal periods. Even though there were 361 exacerbation events, in only 304 of those cases were there at least 7 days immediately before the exacerbation event during which the patient was either in the normal or the transitional state. The remaining cases refer to exacerbation events that occurred within 7 days of a previous exacerbation event. In 18 out of these 304 cases, there was only a single diary session completed by the patient and therefore it was not possible to extract features. This explains why there are 286 prodromal periods extracted from this dataset, from a total of 361 exacerbation events.

**Figure 7:**
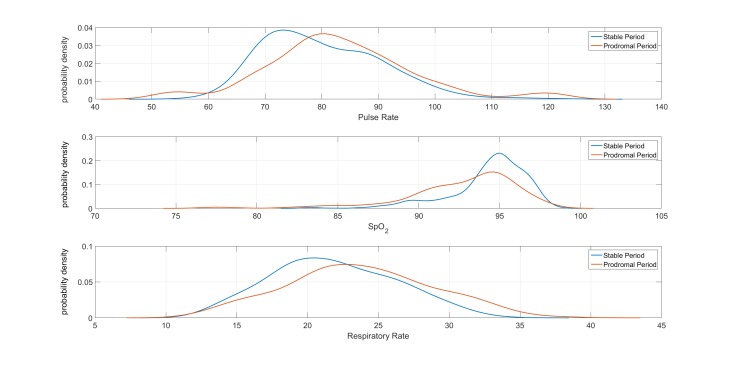
Distribution of vital signs (pulse rate, oxygen saturation or SpO_2_, and respiratory rate) acquired from a pulse oximeter in “stable” and “prodromal” periods.

It can be seen from Table 3 that the mean values for all 3 vital signs are as expected for COPD patients with worsening symptoms: lower mean for SpO_2_ and higher mean for both respiratory rate and heart rate in the prodromal periods in comparison with the stable periods. Figure 7 illustrates the distribution of the 3 vital signs in the stable and prodromal periods. The small difference in the means and the significant overlap between the distribution of the 3 vital signs possibly explain the difficulty of designing algorithms capable of predicting exacerbation episodes.

**Table 3:** Summary statistics of different features in the stable and prodromal periods.

Metric	“Stable” period	“Prodromal” period
Total number of 7-day periods	1005	286
Mean number of sessions in these 7-day periods	5.9	4.6
Mean value of SpO_2_^a^ (%)	94	93
Mean value of pulse rate (beats per minute)	80	83
Mean value of respiratory rate (breaths per minute)	22	24

^a^SpO_2_: oxygen saturation.

### Prediction of Exacerbations

**Figure 8:**
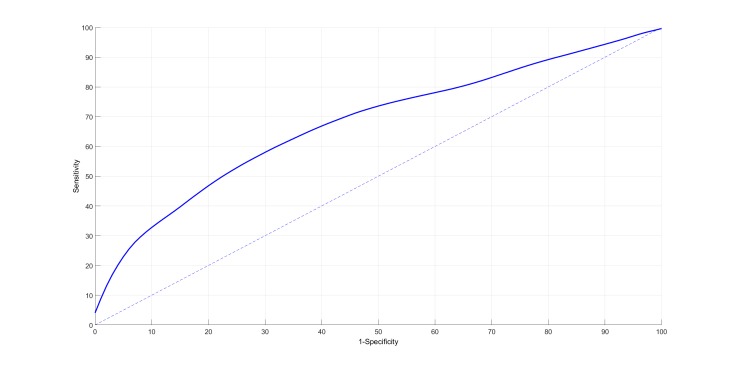
Receiver operating characteristic showing the sensitivity and specificity (mean of 1000 iterations) when using the mean and gradient of pulse rate, SpO_2_, and respiratory rate as input features to the classifiers. Note that the confidence interval is not shown because it is very narrow and the corresponding curves overlap the mean line on the scale shown.

Finally, Figure 8 illustrates the mean ROC curve, bounded by the 95% confidence interval, after applying the logistic classifier to the stable and prodromal periods as explained in the “Classification and Validation” section. Table 4 summarizes the performance results using AUC measures from the mean ROC curve (along with the 95% confidence interval) based on classification with each of the features extracted from each of the vital signs separately and in all possible combinations. In addition, specificity at specific sensitivity measures is also provided to give a better sense of the level of performance that could be expected for a given operating point (threshold) selected from the mean ROC curve. All 3 vital signs have a degree of predictivity because the mean AUC is greater than .5 for all of them. SpO_2_ seems to be the most predictive vital sign, followed by respiratory rate estimated using our algorithm. In comparison with respiratory rate and SpO_2_, pulse rate seems to be the least predictive in this population.

**Table 4:** Comparison of classifier performance using features extracted from each of the vital signs separately and in combination; the sensitivity and specificity values are those extracted from mean receiver operating characteristic curve.

Vital sign	Mean AUC^a^ (95% CI)	Specificity range (%) for 60%-80% sensitivity from mean ROC^b^ curve
Pulse rate (PR)	0.578 (0.578-0.578)	52-31
SpO_2_^c^	0.658 (0.657-0.658)	62-38
Respiratory rate (RR)	0.605 (0.604-0.605)	53-32
PR + SpO_2_	0.664 (0.664-0.664)	63-40
RR + PR	0.612 (0.612-0.612)	55-27
RR + SpO_2_	0.672 (0.671-0.672)	64-36
RR + PR + SpO_2_	0.682 (0.681-0.682)	68-36

^a^AUC: area under the curve.

^b^ROC: receiver operating characteristic.

^c^SpO_2_: oxygen saturation.

The amount of improvement possible as a result of combining vital signs in the classification algorithm can be seen from the two lower rows in Table 4. All 3 vital signs are derived from a single device and have a degree of correlation for physiological reasons. However, it can be seen that classifiers using respiratory rate, pulse rate, and SpO_2_ as inputs give the best AUC results, suggesting that there is more predictive information captured when these vital signs are used together. These results suggest that, in order to predict 8 exacerbations out of 10 (80% sensitivity), there would be approximately 6 false alarms out of 10 (36% specificity). If instead the aim was to predict 6 exacerbations out of 10 (60% sensitivity), this would give rise to approximately 3 false alarms in every 10 (68% specificity).

## Discussion

### Principal Findings

Our digital health system enabled reliable and regular self-monitoring of both symptom and pulse oximetry data by patients with COPD for a sustained period of 12 months. This allowed the acquisition of a unique dataset, with more than 35,000 monitoring days (average of 5.3 times per week). We have also presented a systematic approach to modeling COPD exacerbations based on FSMs. This provides a deeper insight into how a COPD patient’s condition progresses over time. For example, this study showed that in about 97% of the cases for which patients went from the normal state to the transitional state and then back again to the normal state, they did so without increasing their medication. This could be described as normal variation (ie, normal variation in symptoms that does not warrant a change in regular medication). We are not aware of any previous study that has been able to quantify and report this finding from such a large dataset.

A limitation of this study is that the results presented are based on the application of a specific definition of exacerbation. In addition, both the symptom diary information and the medication data used to identify exacerbation episodes are self-reported by the patients. Finally, any exacerbation event occurring while the patient is in a transitional state and contacts an HCP or is admitted to hospital is not identified in our analysis because of lack of availability of this information.

The first step in developing a predictive algorithm is to decouple periods for which a patient’s condition is completely stable from those for which a patient is approaching an exacerbation event (the prodromal period). This paper described a possible approach to the development of a predictive algorithm robust to irregularly sampled data as well as missing data. The features used as inputs to the proposed algorithm were the mean and gradient of each of the vital signs (pulse rate and SpO_2_) acquired by the pulse oximeter or estimated from the pulse oximetry PPG data (respiratory rate) for that 7-day period. A predictive algorithm based on physiological data rather than self-reported symptoms could be really helpful to support self-management in COPD. Pinnock et al [18] report that analysis of previous telemonitoring data suggests that it is the minority of patients who are able to log discrete episodes of increased breathlessness, cough, and sputum.

This study has demonstrated and quantified the predictive power of 3 vital signs (pulse rate, SpO_2_, and respiratory rate) and also shown that they can reliably be obtained in home-monitoring with only a pulse oximeter. This coupled with the fact that only a minority of patients with COPD are able to log episodes of worsening symptoms suggests that the use of a pulse oximeter should be considered in any future study involving remote monitoring of patients with COPD. In addition to extracting respiratory rate, we also explored the possibility of gaining more information from the pulse oximeter waveform (the PPG). A patient in the prodromal period is likely to have a more irregular respiratory pattern, with a greater proportion of power in higher-frequency bands as his or her condition worsens. However, no features associated with the spectral content of the respiratory signal extracted from the PPG provided any further information once the respiratory rate was extracted. We also explored the use of the standard deviation and y-intercept of each of the vital signs in the 7-day periods as additional features, but again this did not lead to improvement in the performance of the predictive algorithm. We also investigated if the classification performance could be improved using support vector machines, decision trees, or k-nearest neighbor–based classifiers [19]. However, none of these algorithms led to any significant improvement with respect to logistic regression. This suggests that further improvements are more likely to come from the use of independent features not explored in this study (eg, temperature, activity monitoring [20], heart rate variability [21], or automated sputum analysis [22]).

We are not aware of any previous work that has proposed FSM or similar models in order to identify COPD exacerbations and track the condition of patients with COPD during remote monitoring. Furthermore, we are not aware of any previous study that has reported respiratory rate measurements from patients with COPD during home monitoring (except one study [23], which only looked at patients requiring domiciliary oxygen, a very specific and narrow segment of the population of patients with COPD, for only 3 months). In this paper, we have reported the distribution of 3 vital signs (pulse rate, SpO_2_, and respiratory rate) during both stable and prodromal periods based on measurements from 100 patients with COPD, providing very frequent measurements over a 12-month period. The majority of previous studies using telemonitoring for COPD were shorter than 12 months [7]. Of the few studies [24-27] that were at least 12 months long, only one [25] had at least 100 patients. However, in that study [25], the alerting algorithm was based on thresholds applied to the symptom score derived from a self-reported symptom diary.

### Conclusions

Given the heterogeneous nature of COPD exacerbations and the large number of definitions in the literature, the FSM-based approach described in this paper can help systematize the analysis of COPD exacerbations and improve our understanding of how symptoms worsen and of the impact of medication.

We showed that all the 3 vital signs (SpO_2_, respiratory rate, pulse rate) are predictive of exacerbation events, with a combination of these vital signs resulting in the best AUC result. SpO_2_ was the most predictive vital sign, followed by respiratory rate, and pulse rate was the least predictive. It is important to think about the trade-off between sensitivity and specificity in this context: false alerts will result in overmedication, but the ability to predict exacerbations may avoid costly hospital admissions.

None of the other classification algorithms (support vector machines, decision trees, and k-nearest neighbor–based classifiers) led to any significant improvement compared with logistic regression. Additional improvements in COPD exacerbation prediction are likely to come instead from the use of additional physiological measures, behavioral measures, and biomarkers (eg, temperature, heart rate variability [21], activity [20], and sputum analysis [22]) as input to the prediction algorithm. The other alternative is to personalize the prediction algorithms over time and adapt the classifier to the range of vital sign values that are typical for that particular individual.

## Abbreviations

AUC: area under the curve
COPD: chronic obstructive pulmonary disease
EDGE: Self Management and Support Programme
FSM: finite-state machine
GOLD: Global Initiative for Chronic Obstructive Lung Disease
HCP: health care professional
PPG: photoplethysmogram
RCT: randomized controlled trial
ROC: receiver operating characteristic
WHO: World Health Organization
